# Pediatric Sarcoidosis With Chronic Massive Splenomegaly and Renal Involvement: A Case Report

**DOI:** 10.1155/carm/1828310

**Published:** 2026-04-24

**Authors:** Golnaz Mobayen, Amirataollah Hiradfar, Azar Dastranji, Masoud Lahouty

**Affiliations:** ^1^ Pediatric Health Research Center, Tabriz University of Medical Sciences, Tabriz, Iran, tbzmed.ac.ir

**Keywords:** infliximab, pediatric, renal failure, sarcoidosis, splenomegaly

## Abstract

**Introduction:**

Systemic sarcoidosis is a rare multisystem rheumatologic disorder that is diagnosed after ruling out other granulomatous diseases. Clinical manifestations and treatment in these pediatric patients are also a challenge. Treatment is typically based on corticosteroids and cytotoxic agents; however, no organ‐specific treatment currently exists. Recent studies have demonstrated the effectiveness of anti‐TNF drugs in treating pulmonary and extrapulmonary sarcoidosis.

**Case Presentation:**

We report the case of a 16‐year‐old boy who, since the age of 12, presented with cytopenia, constitutional symptoms, and abdominal pain without a definitive diagnosis. At Age 16, he developed massive splenomegaly with lung and renal involvement. A diagnosis of systemic sarcoidosis was confirmed via a spleen biopsy. He was treated with infliximab in combination with corticosteroids and a cytotoxic agent.

**Conclusion:**

This report emphasizes the importance of diagnosing sarcoidosis based on rare and nonspecific signs and symptoms and treatment choices. Early diagnosis of this disease can lead to more effective treatment and prevent multiple organ involvement. Although there is not any organ‐specific treatment for systemic sarcoidosis in children, with just a few reports of effectiveness of infliximab in kidney involvement, we decided to treat our case with infliximab because of corticosteroid and cytotoxic failure. This treatment may show that infliximab plays a key role in treating corticosteroid‐resistant sarcoidosis that affects certain organs, like the kidneys, or in reducing the side effects of corticosteroid drugs.

## 1. Introduction

Systemic sarcoidosis is a rare multisystem rheumatologic disorder characterized by noncaseating granulomatous tissue in different organs. Pediatric sarcoidosis is an extremely rare disease, where the incidence is respectively 4.9/100,000/years in adults and between 0.40 and 0.80/100,000/years in children. Children of all ages can be affected, but more than two‐thirds of the patients are over 10 years old at the time of diagnosis [1]. Its prevalence in children is 10 times lower than in adults, and due to the lack of diagnostic criteria in children, it is diagnosed after ruling out other granulomatous diseases, particularly immune deficiencies, granulomatous infections, tumors, eosinophilic granulomatous, Crohn’s disease, and less likely granuloma caused by drugs, along with tissue biopsy confirmation. The most known symptom of this disease is pulmonary involvement. Clinical symptoms can include single‐organ involvement and self‐limited or multiorgan involvement with a high risk and poor prognosis [2]. According to cohort studies of children with sarcoidosis, the most common symptoms are systemic ones, such as fever, weight loss, and tiredness. These are frequently followed by pulmonary, ocular, cutaneous, and articular involvement. Cardiac, neurological, gastrointestinal, and ear, nose, and throat (ENT) manifestations are less common. Treatment remains a challenge in this population [1, 3]. In this study, we describe a 16‐year‐old boy with systemic sarcoidosis that led to massive splenomegaly and presented with cytopenia, splenomegaly, and occasional abdominal pain, without significant pulmonary symptoms despite extensive pulmonary involvement on imaging. He was treated with infliximab, corticosteroids, and a cytotoxic agent.

## 2. Case Report

**FIGURE 1:**
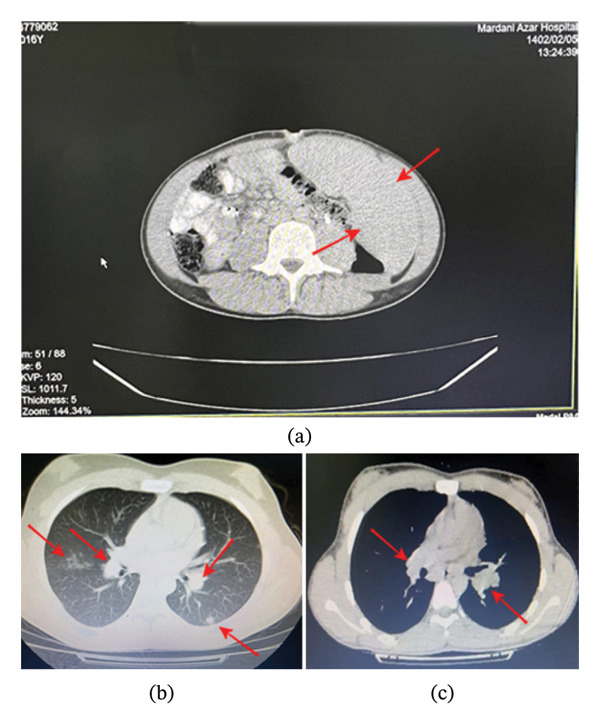
(a) Abdominal CT scan: massive splenomegaly assigned with red arrows. (b) A chest CT scan shows lymphadenopathy on both sides, along with interstitial involvement, subpleural nodules, and per‐fissure nodules (assigned with arrows). (c) Chest CT: bilateral lymphadenopathy (window view).

**FIGURE 2:**
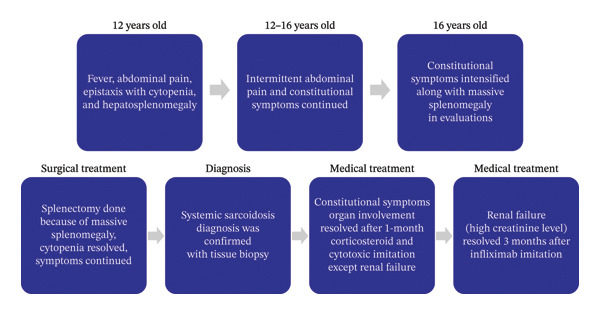
Case progression during 4 years (12–16 years old).

A 16‐year‐old boy with a history of lethargy, anorexia, intermittent fevers, and abdominal pain was advised by a hematology service for a rheumatology consultation. His past medical history was significant for recurrent thrombocytopenia and hepatosplenomegaly since the age of 12, without a specific diagnosis. Physical examination revealed no skin involvement; the musculoskeletal, neurological, and cardiopulmonary examinations were normal. Abdominal examination revealed hepatomegaly with a liver span of 13–17 cm. Due to massive splenomegaly (Figure 1(a)), he underwent a splenectomy, and tissue samples were sent for pathological review. Initial laboratory investigations revealed the following: a white blood cell (WBC) count of 5800/μL (lymphocytes 23% and neutrophils 64%), hemoglobin 15.5 g/dL, platelet count 124,000/μL, erythrocyte sedimentation rate (ESR) 5 mm/h, negative C‐reactive protein (CRP), lactate dehydrogenase (LDH) 701 U/L, negative antinuclear antibody (ANA), negative anti‐dsDNA antibody, vitamin D3 21 ng/mL, serum calcium 10.2 mg/dL, normal liver function tests (LFTs), serum angiotensin‐converting enzyme (ACE) 105 U/L, negative Wright and Coombs Wright tests, and serum creatinine 1.5 mg/dL. Abdominal imaging revealed moderate hepatomegaly and multiple lymphadenopathies in the celiac and para‐aortic areas along with hepatic hypodense areas. Investigations for viral infections, including CMV and EBV, and the level of serum immunoglobulins were normal. Inflammatory markers were within the normal range, and liver enzymes were normal. We performed a bone marrow aspiration and found no evidence of malignancy. On the chest CT scan, there were lymph nodes in both the hilar and paratracheal areas, as well as interstitial involvement in both lungs, subpleural, and per‐fissure nodules (Figures 1(b) and 1(c)). Pathology samples from the spleen and abdominal lymph nodes were suggestive of chronic noncaseating granulomatous inflammation with medium‐sized granulomas without acid‐fast bacilli, in favor of sarcoidosis. A PCR test for tuberculosis on the tissue biopsy was negative. Samples sent to a tertiary hematopathology center showed no evidence of lymphoma or eosinophilic granuloma. Due to elevated creatinine, a kidney MRI was performed, which revealed no pathological findings. The only predominant clinical finding during this period was abdominal pain. The diagnosis of systemic sarcoidosis was confirmed. The patient was treated with pulse methylprednisolone (30 mg/kg/day) for three consecutive days, followed by high‐dose prednisolone (1–2 mg/kg/day) in combination with methotrexate (0.5 mg/kg/week). After 1 month, the serum creatinine level had not normalized; therefore, we initiated monthly infliximab infusions in addition to methotrexate and corticosteroid therapy. The serum creatinine level returned to the normal range after 3 months. Corticosteroids were tapered after 3 months and gradually reduced to a low maintenance dose (0.5 mg/kg/day) within 6 months. A follow‐up chest CT scan at that time showed no mediastinal lymphadenopathy and a significant decrease in nodular and ground‐glass opacities. An abdominal CT scan revealed no lymphadenopathy, and the liver had returned to its normal size (Figure 2 presents the timeline of clinical events during this period).

### 2.1. Learning Points

1. Diagnostic challenges in pediatric sarcoidosis.
2. Treatment innovations with anti‐TNF therapy.
3. Importance of early diagnosis and intervention.

## 3. Discussion

While sarcoidosis in adult patients is often asymptomatic and diagnosed based on radiological findings, histopathological detection of non‐necrotizing granulomas, and the exclusion of other diseases [4], pediatric sarcoidosis typically presents with acute systemic symptoms and multiorgan involvement. More than 70% of children have 1.5–3.8 organs involved, though in rare cases, patients may be asymptomatic [3], and up to five or more organs can be affected [5]. The disease most frequently involves the lungs, lymph nodes, eyes, skin, liver, and spleen in children [6].

According to literature reviews, 60% of children with sarcoidosis have decreased creatinine clearance, but less than 40% of these children exhibit other signs of renal involvement, such as urinalysis abnormalities or elevated blood urea and creatinine levels. Renal damage in these patients is most often attributed to nephrocalcinosis secondary to hypercalcemia or to tubulointerstitial nephritis (TIN), with or without granuloma formation [7]. Although sarcoidosis has an equal gender distribution, males demonstrate a slightly higher prevalence of renal involvement. While a kidney biopsy is required to definitively diagnose renal sarcoidosis, it may be omitted in some cases with a clear diagnosis from other tissues [8].

The most common manifestation of reticuloendothelial system involvement in sarcoidosis is peripheral lymphadenopathy, observed in 40%–70% of cases; hepatosplenomegaly may also be seen in up to 40% of patients. However, the spleen is the predominantly affected organ in only a few cases [9]. Therefore, the presence of massive splenomegaly in our patient is an uncommon presenting sign. Despite pulmonary involvement in most patients, the manifestations are often subtle and usually include a dry cough and chest pain [10]. It is now evident that renal involvement presenting as acute kidney injury (AKI) is a common feature of pediatric sarcoidosis [11].

The most common and effective treatment for systemic sarcoidosis remains corticosteroids. A significant concern, however, is their long‐term complications. Consequently, there is a growing trend toward the use of cytotoxic agents. Methotrexate is the most frequently used and safest of these drugs, followed by agents such as azathioprine, mycophenolate mofetil, and cyclophosphamide in refractory cases. However, the significant toxicity of these drugs limits their long‐term use to patients with frequent relapses [6]. To date, no studies have investigated the use of biologics in children. Recent studies have shown the effectiveness of anti‐TNF drugs in treating pulmonary and extrapulmonary sarcoidosis [12, 13], with the most significant effects reported in patients with chronic cutaneous and neurological manifestations [14]. This therapeutic experience may suggest the important role of infliximab in managing corticosteroid‐resistant sarcoidosis with specific organ involvement, such as the kidney, or in cases where corticosteroid‐related side effects are a major concern.

## Funding

No funding received.

## Ethics Statement

The authors declare that the research presented in this manuscript adheres to the ethical principles outlined by the Ethics Committee of Tabriz University of Medical Sciences, Tabriz, Iran.

All procedures involving human participants were conducted in accordance with the ethical standards of the Ethics Committee of Tabriz University of Medical Sciences and the Declaration of Helsinki (1964), as revised in 2013. Informed consent was obtained from the parents of the patient undergoing treatment, and their privacy and confidentiality were safeguarded throughout the study. Ethics Approval: IR.TBZMED.REC.1403.962.

## Conflicts of Interest

The authors declare no conflicts of interest.

## Data Availability

The data that support the findings of this study are available on request from the corresponding author. The data are not publicly available due to privacy or ethical restrictions.
